# Long-Term Yogic Intervention Improves Symptomatic Scale and Quality of Life by Reducing Inflammatory Cytokines and Oxidative Stress in Breast Cancer Patients Undergoing Chemotherapy and/or Radiotherapy: A Randomized Control Study

**DOI:** 10.7759/cureus.33427

**Published:** 2023-01-05

**Authors:** Mayank Jain, Archana Mishra, Vishnu Yadav, Hari Shyam, Shailendra Kumar, Satyendra K Mishra, Pooja Ramakant

**Affiliations:** 1 Department of Thoracic Surgery, King George's Medical University, Lucknow, IND; 2 Department of Physical Education, University of Lucknow, Lucknow, IND; 3 Department of Center for Advance Research, King George's Medical University, Lucknow, IND; 4 Department of Human Consciousness and Yogic Sciences, University of Lucknow, Lucknow, IND; 5 Endocrine Surgery, King George's Medical University, Lucknow, IND

**Keywords:** immune response, oxidative stress, inflammatory cytokines, yoga, breast cancer

## Abstract

Introduction: Inflammation has been associated with tumor proliferation and metastasis in breast cancer. Yoga is an ancient therapy that helps in reducing inflammation and improves the patient’s quality of life (QoL) and fatigue. In the current study, we investigated the effects of long-term yogic intervention at different time points on the level of inflammatory cytokines and oxidative stress, along with the symptomatic scale and QoL in stage II/III breast cancer patients.

Methods: Ninety-six stage II/III breast cancer patients receiving chemotherapy and/or radiotherapy were enrolled and divided into two groups, non-yoga (Group I) and yoga (Group II). Participants in Group II practiced yoga five days per week for 48 weeks. The European Organisation for Research and Treatment of Cancer quality of life questionnaire (EORTC-QLQ30) was used to measure the QoL and symptomatic scale. Serum levels of pro-inflammatory cytokines, tumor necrosis factor-alpha (TNF-α), interferon-γ (IFN-γ) and granulocyte macrophage colony-stimulating factor (GM-CSF), and oxidative stress markers, superoxide dismutase (SOD), catalase (CAT), malondialdehyde (MDA), and nitric oxide (NO) were measured at baseline, 16, 32, and 48 weeks in both groups.

Results: Yoga significantly (p<0.05) reduced the level of IFN-γ, TNF-α, and MDA and improved QoL (p<0.001) and symptomatic scale (p<0.05) in Group II patients compared to Group I. NO was upregulated in Group I whereas in Group II, it was neither decreased nor increased.

Conclusion: These findings suggest that yoga may reduce levels of inflammatory cytokines and improve QoL and symptomatic scale in breast cancer patients receiving chemotherapy and/or radiotherapy. Yoga can be an important additional therapy during cancer treatments to cope with treatment side effects including fatigue, depression, and immunological profile, which directly affects the patient’s quality of life.

## Introduction

Globally, breast cancer is a leading cancer in women and the major cause of death among cancer patients [1]. Tumor necrosis factor-alpha (TNF-α) and interferon-γ (IFN-γ) are the most studied pro-inflammatory cytokines in breast cancer. These are associated with chronic inflammation and have an important role in tumor development [2]. Interferons (IFNs) are cytokines that regulate molecular, cellular, and physiological processes during inflammation [3]. IFNs can influence cancer cell metabolism and proliferation through a variety of molecular pathways [4]. Dendritic cell production can be accelerated by granulocyte-macrophage colony-stimulating factor (GM-CSF). GM-CSF is upregulated in breast cancer patients and promotes angiogenesis.

The inflammatory process is often linked to oxidative stress, and it can act as a signal to the inflammatory process or vice versa [5]. Malondialdehyde (MDA) is the major lipid peroxidation byproduct and served as a marker for oxidative stress [6]. Physiologically, the superoxide dismutase (SOD) family is crucial in reducing the negative effects of ROS. Decreased catalase (CAT) activity is also associated with several cancers. Nitric oxide (NO) is important in both development and suppression of tumorigenesis, which depends on its origin and concentration. Increased oxidative stress has been reported in the manifestation of many diseases [7]. The balance between the anti-oxidants and pro-oxidants is essential for the metabolic process [8]. This imbalance leads to activation of regulatory genes of chronic inflammation. Breast cancer treatments increase inflammation and oxidative stress in patients [9]. Cancer treatments often cause physical problems (fatigue, pain, dry mouth, insomnia, nausea, vomiting) and psychological problems (emotional distress, anxiety, depression) in patients [10].

Yoga has been practiced for many years and has been shown to be an effective adjuvant therapy for various conditions including diabetes, asthma, acquired immune deficiency syndrome (AIDS), heart failure, immunological function, serum cortisol levels, and lymphoma [11]. Several studies have found that practicing yoga on a regular basis reduces the frequency of natural killer cells and DNA damage during chemotherapy [12]. Yoga has been beneficial in reducing oxidative stress in prostate cancer, type 2 diabetes and Parkinson’s disease [13,14]. Yoga has been beneficial for breast cancer survivors by improving functional, physical, and emotional well-being. Studies have also suggested that physically active patients have a lower risk of cancer recurrence and mortality [15]. 

In previous studies the primary outcome of yoga on breast cancer survivors was QoL parameters. Previous yoga studies related to oxidative stress were limited to diabetes, stress, and some other diseases. These studies suggested that yoga effectively reduces the level of oxidative stress markers such as MDA, SOD, NO and catalase [14]. Literature related to breast cancer was very limited and duration of the intervention was between one to six months. Hence, we designed a randomized control study to observe the effect of the long-term yogic intervention on the level of oxidative stress and inflammatory cytokines in breast cancer stage II/III patients undergoing chemotherapy and/or radiotherapy.

This data has been presented as a poster at the European Breast Cancer Conference (EBCC-13) conference from 16th to 18th November 2022 and ESMO-Asia 2022 congress from 2nd to 4th December 2022. The abstract of the presented poster was published in a supplement of the European Journal of Cancer and Annals of Oncology.

## Materials and methods

Subject selection

The institutional ethics committee of King George's Medical University approved this study (111th ECM IIB-Ph.D./P3). Patients were evaluated according to the inclusion (age between 30-65 years, stage II/III, undergoing radiotherapy and/or chemotherapy, fit for yogic interventions) and exclusion criteria (pregnant females, suffering from major gynecological disorder, second malignancy, on other alternative medicine, not willing to participate in the study, co-morbid illness like a cardiac disease, hepatic disorder, older than 65 years). Patients were enrolled after providing written information consent. Sample size was calculated using the prevalence (GLOBOCAN 2020), error, degree of freedom [1]. Calculated sample size was 42 and 15% extra has been taken for dropouts. Therefore final sample size in each group is 48.

A total of 96 stage II/III breast cancer patients undergoing chemotherapy and/or radiotherapy were enrolled from the outpatient department (OPD) of Endocrine Surgery and Surgery. Patients were equally divided using the double-blind (envelop-based) randomization method into two groups. Group I (non-yoga) patients received only conventional treatment (chemotherapy and/or radiotherapy) whereas Group II (yoga) received additional yogic intervention five days/week for 48 weeks. We measured the levels of oxidative stress markers SOD, CAT, MDA, NO, and inflammatory cytokines TNF-α, IFN-γ, and GM-CSF along with the quality of life and symptomatic scale questionnaires at different time points (baseline, 16, 32, and 48 weeks) in both groups.

Yoga session

Patients in the yoga group were trained by the yoga teacher and monitored until they performed the yoga. The details of these yogic asanas are: Sukshma Vyayama (loosening exercises) (seven minutes), Tadasana (mountain pose) (two minutes), Kati Chakrasana (lateral arc pose) (two minutes), Padadhirasana (standing forward bend) (three minutes), Tiryaka Tadasana (swaying palm tree pose) (two minutes), Hridaya Mudra (heart gesture) (two minutes), Gomukhasana (cow face pose) (three minutes), Nadi Shodhana Pranayama (alternate-nostril breath) (three minutes), Bhramari Pranayama (bee breath) (two minutes), Ujjayi Pranayama (Ocean Breath), and Shavasana (corpse) (five minutes). Humming in meditative posture: meditation, Om chanting (10 minutes), or Yoga Nidra (alternate days for 30 min) [16].

During the treatment, subjective and objective assessments were held every month, and homework was monitored on a day-to-day basis through video calls. Patients were asked to keep their daily log and communicate with us. Later, it was recommended that they perform the same activity five days/week at home. Weekly practice and session forms were obtained and monitoring of the yoga session was done through phone and video calls. The recommended yoga practices were followed by the patients. Group I patients were told not to practice any type of yoga during the study.

Quality of life (QoL) and symptomatic scale

EORTC-QLQ30, the validated questionnaire of the European Organisation for Research and Treatment of Cancer, was used to measure the quality of life and symptomatic scale. For symptomatic scale total 13 questions related to fatigue, nausea, vomiting, pain, dyspnoea, insomnia, appetite loss, constipation, diarrhoea, financial difficulties were recorded with a scale from one to four. Higher the % of quality of life and lower % of symptomatic scale suggested improvement. The questionnaire was filled out independently by all patients who were able to read and understand the Hindi/English language and other patients were given help filling out the questionnaire. Data of the symptomatic scale and quality of life were normalized and calculated according to the EORCT protocol.

Sample collection and serum isolation

All participants' blood samples were collected at baseline, 16, 32, and 48 weeks in clot vials which were kept at room temperature for 30 minutes. Tubes were centrifuged for 30 minutes at 1500g, 4°C and serum was collected in 1.5 ml tubes and stored at -80°C till further analysis.

Thiobarbituric acid reactive substances assay (TBRSA)

100µl serum and quantitative standards were collected in separate tubes, 200 μL of 8.1% sodium dodecyl sulfate (SDS) was added then the solution was gently swirled. Then 1.5 mL of 3.5 M sodium acetate buffer (pH = 4) and 1.5 mL of aqueous 0.8% thiobarbituric acid (TBA) solution (pH = 4) was added and make up the final volume using 700μL of deionized water. Tubes were incubated first at 95°C on the water bath for one hour and then on ice for 30 minutes. Tubes were centrifuged for 10 minutes at 1500 g, 4°C. Absorbance were read at 532 nm [17].

Determination of serum NO level

The Griess reagent was used to determine the NO level. A serum sample (40 µL) and standard were added into a 96-well plate, then 160 μL of Griess reagent was added and kept at room temperature for 20 minutes. The absorbance at 540 nm was read by using a spectrophotometer (Molecular Device Corp., Sunnyvale, CA, USA) to detect the purple-azo-dye product [18].

Superoxide dismutase (SOD) assay

SOD was determined using the pyrogallol autoxidation method. 50 μL of serum and the standard were collected in a glass tube, and 1 mL of Tris buffer (pH = 8.2) was added to the sample and mixed briefly then pyrogallol was added and the absorbance read at 420 nm after one minute. The autoxidation of pyrogallol in the presence of EDTA at a pH of 8.2 is 50% [19].

Catalase (CAT)

CAT was determined using a colorimetric method [20]. In a test tube, 100 μL of serum was collected along with 1 ml of 0.01 M phosphate buffer (pH 7) and 400 μL water. The reaction was started with the addition of 500 μL of H2O2. All the tubes were incubated at 37°C for one minute. Then 2 mL of potassium dichromate-acetic acid reagent (1:3 ratio) was added to stop the reaction. After 15 minutes in a boiling water bath, the tubes were cooled and the absorbance at 570 nm was measured against the control [18].

Cytokine estimation

Serum cytokines were assayed by the Human Cytokine Magnetic 10-Plex Panel (Invitrogen, Waltham, MA, USA) using the MAGPIX instrument (Luminex, Austin, TX, USA). It was used to determine the concentrations of pro-inflammatory cytokines (IFN-γ, TNF-α and GM-CSF) according to the manufacturer’s instructions manual. Inter-assay variation (%) of the cytokines was TNF-α (8.3%), IFN-γ (9%), and GM-CSF (9.1%). According to the manufacturer, the assay sensitivity (pg/mL) for the cytokines GM-CSF, IFN-γ, and TNF-α is less than 0.5. 

Statistical analysis

Categorical variables are represented in the form of percentages, and continuous variables were represented as mean and standard deviation (SD). For the oxidative stress and inflammatory markers, the concentration was calculated against the standard curve and linear regression equation. The Kruskal-Wallis one-way analysis of variance test was done as appropriate to analyze the data at different time points and compare all the groups. To determine the inter-group significance, a two-way ANOVA was used. A p value of <0.05 was considered statistically significant.

## Results

Patient classification

Ninety-six breast cancer patients were enrolled in this study and divided randomly into two groups (48 patients in each group). Out of which five patients in Group I and two in Group II died, whereas three patients in Group I and four in Group II were lost in follow-up at various points. No other problems due to the yogic intervention were reported during the follow-up. The mean age of the patients in Group I was 47.67±11.68, and in Group II, 43.11±9.39. Approximately 70% of patients in both groups had infiltrating ductal carcinoma, and the other 30% had other types.

Socioeconomic and demographical status

Ninety percent of the patients in both groups were from the lower to lower-middle socioeconomic class. In both groups, 70% of the patients were from rural areas. All the patients were married, and out of them, 3% in control and 8% in yoga were widowed. The majority of the patients in each group were illiterate, 69% in the control group and 54% in the yoga group. More details of the clinical characteristics, socioeconomic status and demographics of the study population were published in a previous article [16].

Patient-reported symptomatic scale and QoL

**Figure 1:**
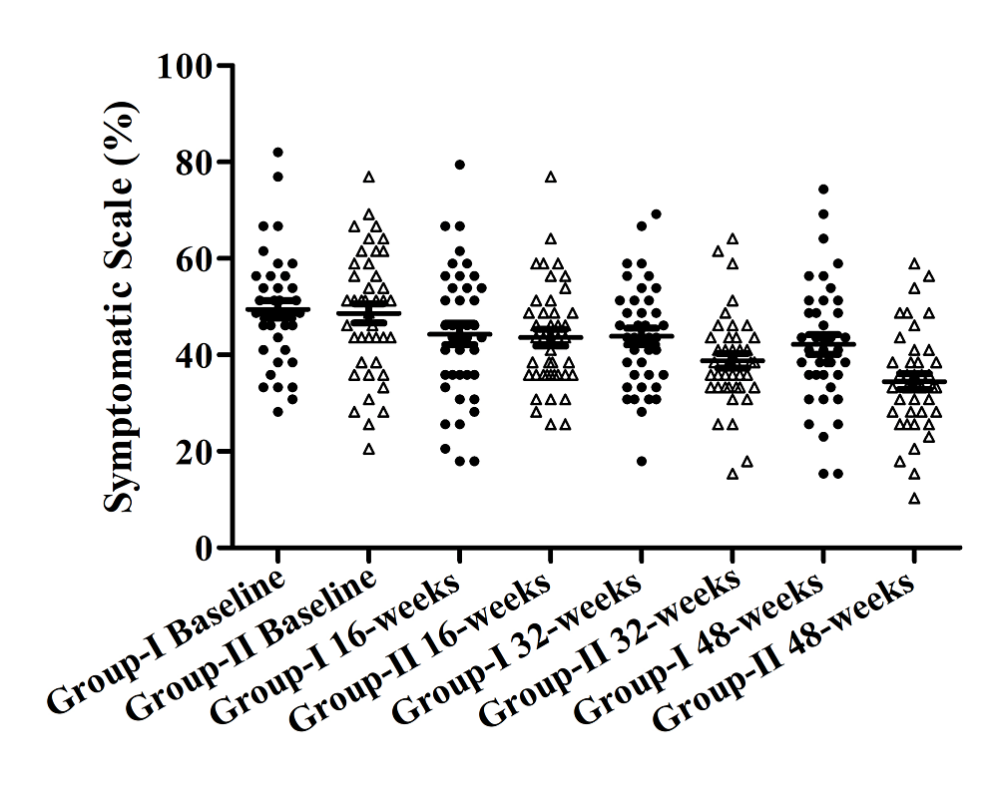
Symptomatic scale in both groups

Within Group II symptomatic scale decreased from the baseline to 16, 32, and 48 weeks were 48.66±1.99, 43.59±1.69, 38.77± 1.49, and 34.49±1.62 respectively. The most significant difference was found between the baseline and 48 weeks within Group II. Within Group I decrease from baseline to 48 weeks was also recorded, but the difference was not significant (Figure 1).

**Figure 2:**
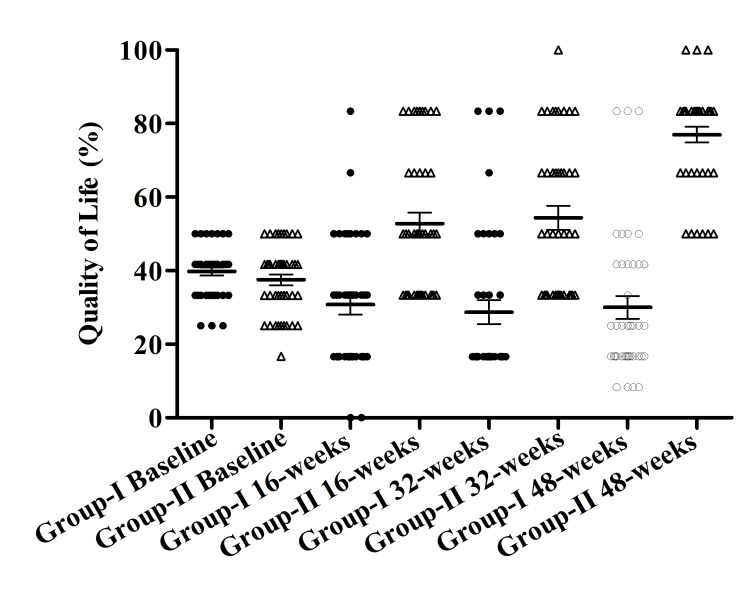
Quality of life in both groups

Within Group II QoL increased from the baseline to 16, 32, and 48 weeks were 37.50±1.45, 52.78±3.00, 54.37±3.21, and 76.99±2.12 respectively. A significant difference was found between the baseline and 48 weeks within Group II. Whereas within Group I QoL decreased from baseline to 48 weeks, 39.79±1.09, 30.83±2.77, 28.75±3.27, and 30.00±3.15 (Figure 2).

Effect of yogic intervention on the level of MDA

**Figure 3:**
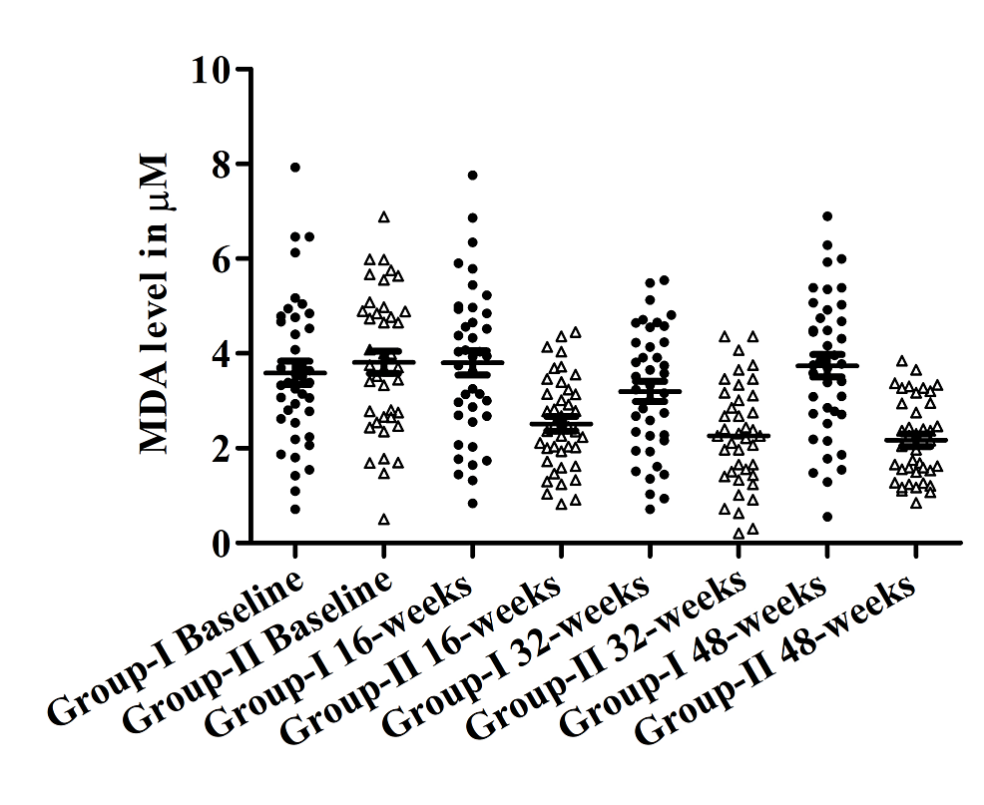
Malondialdehyde (MDA) (µM) levels in both groups

A significant reduction was found in the level of MDA between Group II vs. Group I. Serum MDA levels were downregulated significantly over the period within Group II from baseline to 16, 32, and 48 weeks 3.81±0.23, 2.51±0.15, 2.27±0.17 and 2.167±0.13 respectively. Whereas serum MDA levels within Group I were slightly increased from baseline 3.59±0.24, to 3.80±0.25, 3.2±0.20, and 3.74±0.23 at 16, 32, and 48 weeks. The most significant difference was obtained between Group I vs. Group II at 48 weeks (3.74±0.23 vs 2.16±0.13) (p<0.001) (Figure 3).

Effect of yogic intervention on the level of NO

**Figure 4:**
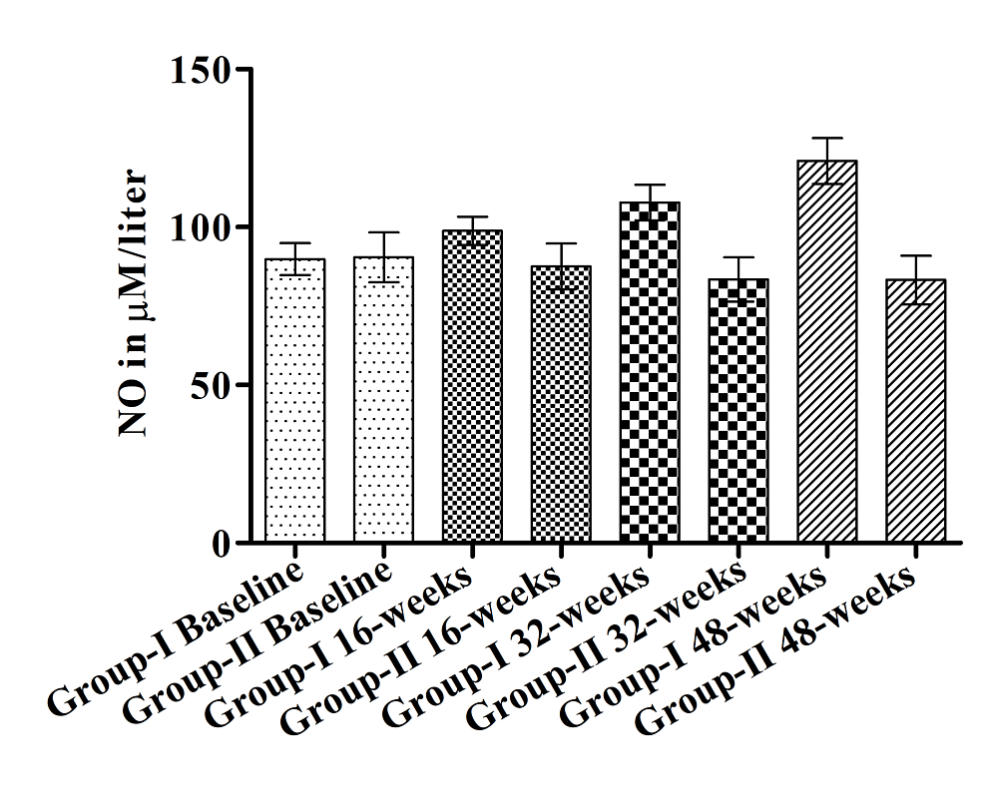
Level of nitric oxide (NO) (μM/liter) in both groups

A significant difference was observed in the level of NO between Group II and Group I at 48 weeks (121±7.25 vs 83.26±7.69). Serum NO level was increased gradually within Group I during the treatment from baseline to 16, 32, and 48 weeks (89.85±5.09, 98.85±4.49, 107.8±5.59, 121±7.25 respectively). Yoga reduced the NO level within Group II from baseline (90.41±7.85) to 16 weeks (87.52±7.26), 32 weeks (83.46±6.97) to 48 weeks (83.26±7.69) but the difference was not significant (Figure 4).

Effect of yogic intervention on the level of antioxidants SOD and CAT

There were no significant differences found in SOD and CAT levels in either group.

Effect of yoga on the level of TNF-α

**Figure 5:**
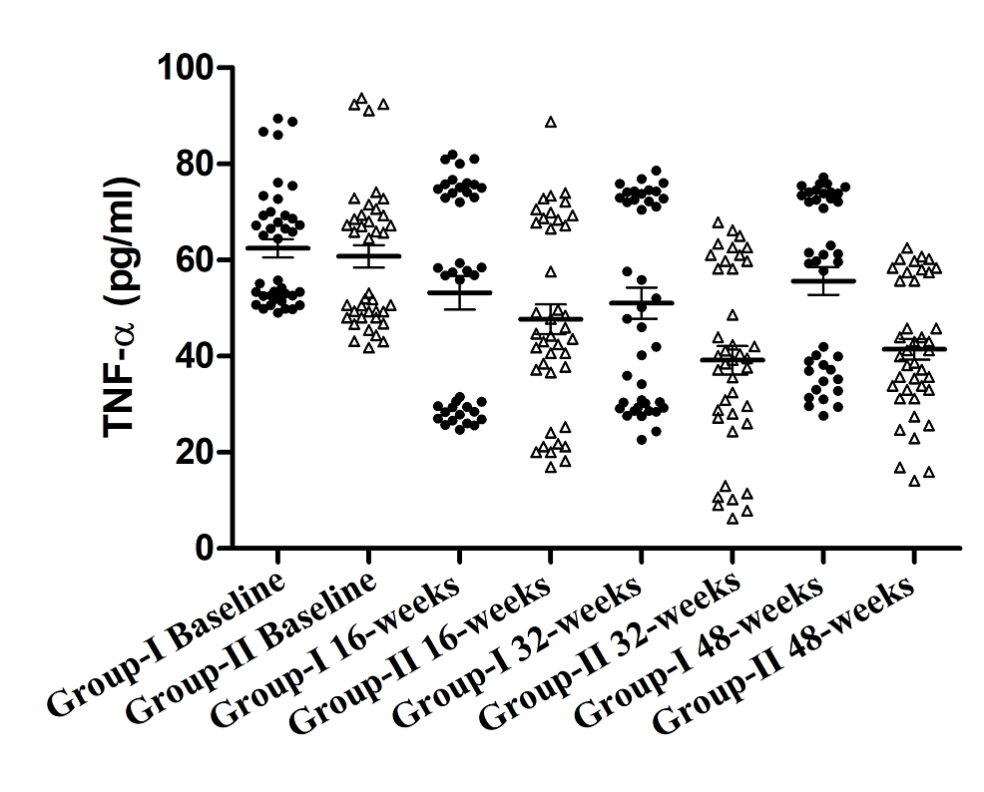
Tumor necrosis factor alpha (TNF-α) (pg/ml) in both groups

Significant change within Group II was observed in the level of TNF-α from baseline to 16, 32, and 48 weeks from 60.79±2.34, to 47.7±3.09, 39.2±2.95, 41.43±2.17. Within Group I downregulations in the level of TNF-α were observed from baseline to 16 and 32 weeks 48 weeks 62.47±1.91, 53.22±3.49, 51.05±3.25, 55.65±2.86. But the downregulation within Group I was not significant. Whereas the between-group analysis showed the most significant difference between Group II vs. Group I at 32 weeks and 48 weeks (Figure 5).

Effect of yoga on the level of IFN-γ

**Figure 6:**
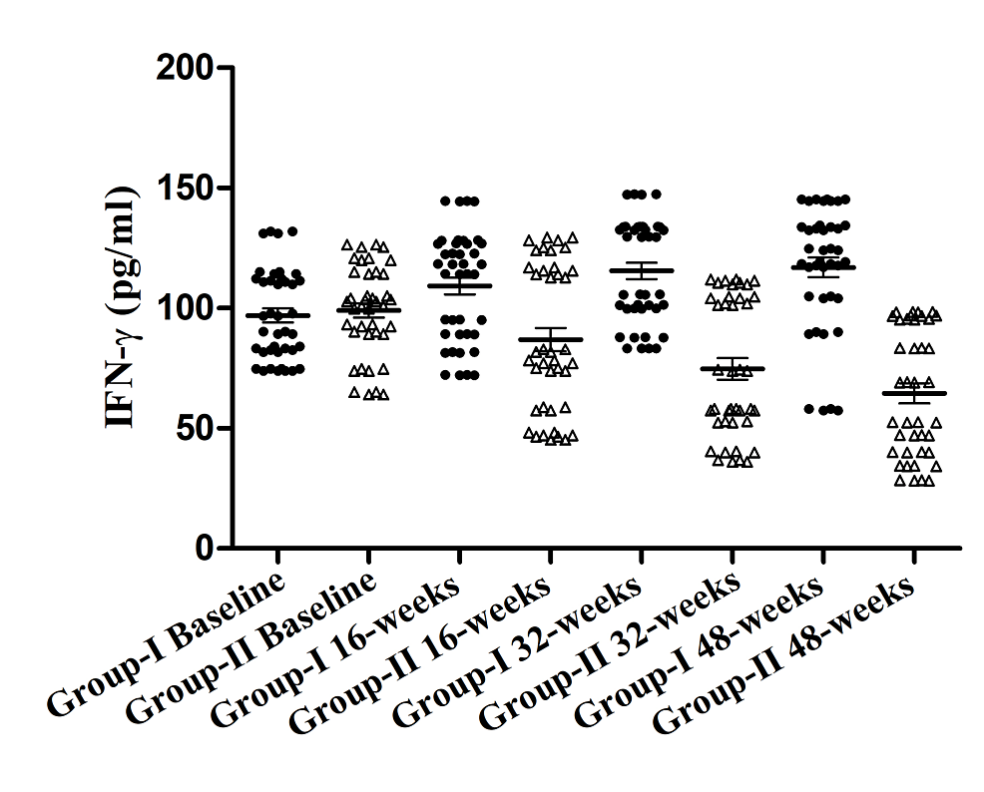
Interferon-γ (IFN-γ) (pg/ml) in both groups

Significant changes within Group II were observed in the level of IFN-γ (pg/ml) from baseline to 16, 32, and 48 weeks 99.05±4.23, 86.93±6.91, 74.7±6.47, 64.55±5.98 respectively. Whereas significant upregulation was found in the level of IFN-γ (pg/ml) within Group I from baseline to 16, 32, and 48 weeks 96.98±4.22, 109.3±5.09, 115.5±4.88, 117.0±4.11 respectively. Intergroup analysis showed the most significant difference between Group II and Group I at 48 weeks (Figure 6). 

Effects of yoga on the level of GM-CSF

There was no significant difference found between or within groups.

## Discussion

In this study, we enrolled stage II/III breast cancer patients and measured the levels of pro-inflammatory cytokines (TNF-α, IFN-γ, GM-CSF) and oxidative stress markers (antioxidant enzymes: SOD and catalase, pro-oxidant: MDA and NO) in the serum along with QoL and symptomatic scale at baseline, 16, 32, and 48 weeks and compared them with patients who were not performing the yoga. Our results showed that yoga improved QoL and symptomatic scale in Group II over time. Yoga showed a significant reduction in the level of pro-inflammatory cytokines (TNF-α, IFN-γ) and pro-oxidant (MDA and NO) in Group II. However, no significant difference in the levels of antioxidant enzymes (SOD and CAT), and inflammatory cytokine (GM-CSF) were observed.

Various studies have reported that yoga has been very beneficial for breast cancer survivor. In these studies, the duration of yogic intervention was between one month to six months [21] and in this study we have given yoga for 48 weeks which is higher than the reported studies. Study showed that four months of yoga intervention improves the quality of life [22]. Systemic analysis showed that yoga improved the inflammatory response in the cancer survivor. Upregulation of the TNF-α was associated with poor quality of life and poor symptomatic scale in breast cancer patients [23]. Some studies measure the effect of yoga on the concentration of inflammatory cytokines (IL-6, IL-8 CRP, TNF-α) and did not find any significant difference [24]. The sample size of these studies was small and the duration of the yogic intervention was six months. But our studies showed the most significant difference at 48 weeks, which suggests the importance of long-term intervention. Increased expression of TNF-α is associated with many cancers, most importantly with breast cancer. It is also correlated with metastasis and a poor prognosis for the patients [25]. Several studies have found that the level of TNF-α is reduced in response to chemotherapy and that it correlates with treatment response [2,26]. A recent study showed that 12 weeks of yoga reduced TNF-α levels in breast cancer patients [27]. INF-γ was found to be higher in breast cancer patients than in healthy people in studies [28]. In many diseases, INF-γ concentration was reduced after yogic intervention [29]. Our data also suggested that yoga improves the level of INF-γ in the patients performing yoga.

Studies also reported that the level of MDA was upregulated in many cancers (breast, ovarian cancer, gastric and lung cancer, and colorectal adenomas) and suggested that it’s involved in the etiology and progression of breast cancer [30]. Previous research has found that breast cancer patients have higher MDA levels than benign breast cancer patients or healthy controls [31]. Yoga reduces the MDA concentration in many diseases such as diabetes, prostate cancer, depression, etc. [32]. But the effect of long-term yogic intervention on the MDA concentration was not studied in breast cancer patients. Our study showed that yoga gives the most significant improvement at 48 weeks.

NO concentration has been evaluated in many cancers. NO has both tumoricidal and tumor-promoting effects, depending on when, where, and how much it is produced. On the other hand, it is showing promise as an anti-oncogenic agent. Inflammation within the tumor microenvironment is thought to be linked to increased invasiveness and a poor prognosis in breast cancer [33]. Some studies reported that levels of NO were significantly upregulated in breast cancer patients compared to controls. Yoga has been beneficial in reducing the level of NO in type 2 diabetes. But the effect of yoga on the level of NO was not studied in breast cancer patients during chemotherapy and/or radiotherapy. Our data suggested that NO concentration was upregulated in the patients not performing yoga, but in the yoga group it was neither increased nor decreased.

The study had some limitations: it was a single-centre study, the sample size was relatively small, and subgrouping of the patients was not done.

## Conclusions

Our current findings showed that long-term yogic intervention improves the quality of life and symptomatic scale and also promote immune response in breast cancer patients by lowering inflammatory cytokines and oxidative stress. In the management of cancer-treatment-related side effects, yoga asanas can be as considered a non-invasive adjuvant therapy. Further studies are needed to validate these findings in a larger population with inclusion of translational component to investigate the effects of yogic intervention.
